# Regarding an “Almost Anything Goes” Attitude Toward Methods in Psychology

**DOI:** 10.3389/fpsyg.2021.612570

**Published:** 2021-02-19

**Authors:** Steffen Zitzmann, Lukas Loreth

**Affiliations:** ^1^Hector Research Institute of Education Sciences and Psychology, University of Tübingen, Tübingen, Germany; ^2^Department of Psychology, Kiel University, Kiel, Germany

**Keywords:** methodology, statistics, disapproval, respect, tolerance

“The beauty of the universe consists not only of unity in variety, but also of variety in unity.”

– Umberto Eco

Post-modernism is a movement that recognizes and encourages different views. As such, it has shaped contemporary democratic societies. One key feature of these societies is diversity, which enriches such societies through cultural exchange and learning. However, because diversity can also lead to conflicts, there have been calls for mutual tolerance (Scanlon, 2003; Popper, 2013). In recent years, psychology has increasingly acknowledged the notion of tolerance as the attitude that one permits others to have different ways of life (i.e., their beliefs, preferences, and practices) despite one's disapproval of them (e.g., Simon, 2020; see also Verkuyten et al., 2020). Tolerance is made possible by respecting others regardless of one's disapproval of them; i.e., by recognizing them as equals (e.g., as citizens with the same rights, duties, and liberties). It is the basis for peace and also paves the way for cooperation between people across boundaries.

In this commentary, we assume that contemporary psychology can be characterized in the very same way as democratic societies. Psychology encompasses not only a variety of different subdisciplines but also proponents of different statistical approaches and methods. These researchers often disapprove of one another because they disapprove of other researchers' work, specifically the statistical approaches other researchers adhere to or the particular methods they prefer. Note that the term disapproval refers to disapproval of what these researchers stand up for. Thereby, it taps the essential, defining feature (e.g., being a Frequentist vs. being a Bayesian) and not some sort of interpersonal disliking. To exemplify such mutual disapproval among researchers, we point to two controversies that are currently heating up and that serve as examples. We then briefly discuss the impact of such controversies on research practice on the basis of our own experience as researchers in the field. Finally, as a remedy, we offer a post-modern methodology that is liberal, pluralistic, and more tolerant.

Today, two principal ways of doing statistics are currently in use in psychology. In the early days of modern statistics, the Bayesian approach—named after Thomas Bayes—took shape and played a major role in the field of statistics, whereas later in the 20th century, it was superseded by the frequentist approach, which was launched by Ronald A. Fisher and others and which many researchers have adopted in their work. Although this approach dominates in psychology, the Bayesian approach has been on the rise again in recent years (van de Schoot et al., 2017). The main difference between the two approaches, and also often the point of disagreement among researchers, is that the Bayesian approach uses not only the data at hand but also a so-called prior distribution. The prior expresses previous knowledge from a previous study, a meta-analysis, or an expert, for example. Conversely, the frequentist approach does not make use of such information, and this has led Efron (2005) to compare a researcher who adheres to the frequentist approach with “a Bayesian trying to do well, or at least not too badly, against any possible prior distribution” (p. 2). The Bayesian approach has many advantages. Among them, perhaps the two most interesting ones are the abilities to incorporate previous findings from related studies and to stabilize models by appropriately specifying the prior (Lüdtke et al., 2018; Zitzmann et al., 2020; see also Zitzmann et al., 2021). For further arguments for why the Bayesian approach might be attractive to researchers, see, e.g., Muthén and Asparouhov (2012) or Depaoli and Clifton (2015).

Besides the major controversy regarding the “right” approach to statistics in psychology, there are also minor controversies. One such controversy is related to the validity of different methods for estimating measurement models that differ in their assumptions and procedures. Specifically, researchers have held debates on which approach is most suitable: factor-based or composite-based methods. Although factor-based methods, such as common maximum likelihood factor analysis or structural equation modeling tend to dominate in psychology, proponents of composite-based methods have argued that these methods can be superior to factor-based methods. One such composite-based method is partial least squares (PLS), and scholars have emphasized the advantage of PLS when the sample size is small (Wold, 1982). This is because the method does not fit the whole model at once but first divides the model into simpler submodels, and then these submodels are fit separately (Tenenhaus et al., 2005; Zitzmann and Helm, 2021). See Rigdon et al. (2017) for an in-depth discussion. However, the fact that the Bayesian approach and PLS can be advantageous under certain conditions does not mean that the frequentist approach and the factor-based methods should be abandoned. Rather, we want to acknowledge that all methods have their undeniable strengths and all are indeed useful in practice.

Much of our motivation for writing this commentary has stemmed from our own experience in getting articles published as well as from the many discussions we have had with other researchers in the field. We believe that readers have similar experiences, although there may be nuances. Publishing an article that uses non-standard methods is still challenging because journal editors and reviewers tend to be overly critical, particularly when they favor another method. Moreover, researchers tend to overlook or even actively ignore even the published work of other researchers when this work used a different statistical approach or method. All of this hampers scientific progress, and we think researchers can and *should* do better.

Karl Popper, who is well-known by psychologists, suggested that science should be “hypothetico-deductive,” meaning that researchers should scrutinize theories by deducing and then testing hypotheses that are falsifiable on empirical grounds. Not all but a great deal of research in psychology is devoted to this idea, and research articles are usually framed in this way. However, in his famous book *Against Method*, Paul Feyerabend (2010) argued that the prescription of one method could hamper science and that the spectrum of methods is much broader. He suggested that science would benefit from a mild “anarchism” (i.e., no rigid rules), which is why he was called an “anarchist.”^1^ We use the word “liberalism” here instead of “anarchism” because we find it more suitable for characterizing our concept of a post-modern methodology. Moreover, and more important for our proposal, Feyerabend also coined the phrase “anything goes,” which we also use and with which we refer to a more pluralistic methodology. By this, we mean that adhering to a specific statistical approach or using a specific method in research practice is perfectly fine, but there are more approaches and methods out there, and researchers should tolerate the researchers who use these methods.

In recent years, the concept of tolerance has been developed in social psychology by adopting ideas from philosophy (e.g., Honneth, 1995; Forst, 2013), and this has led to the disapproval-respect model of tolerance (Simon and Schaefer, 2016; see also Simon et al., 2019; Simon, 2020). This model is a dual-level model. In accordance with Turner et al. (1987), the model assumes that social groups and their respective identities are hierarchically arranged. Members of different groups at a lower level can be members of the same superordinate group at a higher level. A common group identity at the higher level grounds a mutual recognition of equality, meaning that members of different subordinate groups can still recognize each other as equals (because they belong to the same group at the higher level). This ability has also been termed “respect for others as equals.” Respecting others as equals is the main driving force for tolerance, whereas disapproval is a definitional condition for tolerance (Simon, 2020). This means that to develop mutual tolerance, people do not need to give up their disapproval (or their lower level identity). Rather, they need to be embedded in the shared superordinate identity and respect each other as equals (Simon, 2020).

This model can be applied to psychology as well. Researchers are capable of respecting other researchers as equal fellow researchers, even when they differ in their work, specifically in the statistical approaches they adhere to and the methods they use. Respect may then help researchers develop mutual tolerance (i.e., the attitude that one permits the different approaches and methods used by other researchers despite one's disapproval of them) because respect drives tolerance. It is important to note that recognizing equality does not require researchers to see similarities between different approaches or methods or require them to start liking other researchers. The task is much simpler: They need to strengthen their shared identity as psychologists. One way to further strengthen this identity is to facilitate publication in the same respected journals even for researchers whose approaches and methods are not mainstream. To this end, editors should be aware of whether their own as well as the reviewers' methodological critiques are really justified or whether they are merely an expression of dislike. To help editors, they could—if not should—select reviewers who are also familiar with non-mainstream methods. Once they are publishing in the same respected journals, researchers might become aware of the work of others and might even be influenced by such work. We would like to note that the evaluation of possible shortcomings needs some kind of standard. Whether this standard must be a common standard or whether each approach or method can only be evaluated against its own standards can be debated. We think that a common standard is not incompatible with our proposed methodology because different methods can nevertheless be evaluated and compared with each other by applying general criteria, such as statistical criteria (e.g., the accuracy of results).

Another way to strengthen researchers' shared identity is to change the way University teachers teach methods. We suggest they always be taught “in the plural” (see Gigerenzer et al., 2004), which means that non-mainstream methods should also be an element of the method curriculum. However, most University teachers are not trained in non-mainstream methods, and psychology textbooks do not show and explain these methods. As a remedy, University teachers might want to consult the literature on data science, a discipline that is intended to understand phenomena using data and a plethora of different methods to analyze them.

In sum, this commentary points out two controversies that surround methods, followed by a brief outline of a post-modern methodology in psychology. We view mutual tolerance among proponents of different statistical approaches and methods as the key feature of this methodology, which is vital for a pluralism of methods and thereby also for psychology in post-modern times because it facilitates fruitful exchange and cooperation.

We wish to emphasize that such a methodology does not necessarily imply pragmatism. Pragmatists sometimes argue that the significance of the statistical approach or method is often exaggerated because, in many applications, the practical differences between numerical results are only small, and thus, results can be interpreted in multiple ways (e.g., Albers et al., 2018). However, we think that even if results are very similar, their interpretations will nevertheless differ, and researchers may want prefer one or the other of these (different) interpretations. Therefore, we suggest that interpretations not be mixed or blurred but be in accordance with the specific approach or method that was used (see Nalborczyk et al., 2019, for a very similar argument). Moreover, our post-modern methodology does not imply that truth cannot be approached or constructed. We view the search for truth (or the construction thereof) as a guiding principle for researchers that operates as a further source of a mutual recognition of equality: Recognizing this task as a collective endeavor can help researchers give respect to others as equal fellows and tolerate them even when they disapprove of them. Also, our notion of post-modernism does not entail that any discourse is acceptable. We indicated this by the word “almost” in the phrase “almost anything goes” in the commentary's title. This means that we do not consider a radical epistemological critique. Rather, we suggest that the discourse should take place with adherence to a minimal standard (e.g., statistical criteria). First and foremost, our notion entails a call for mutual tolerance, i.e., a critique of rejecting methods because they do not conform to the dominant ideology.

To conclude, our outline points out three aspects of a new post-modern methodology in psychology: liberal, pluralistic, and more tolerant: liberal because it rejects rules that are too strict in favor of more freedom in the choice of method, pluralistic because it conveys an “almost anything goes” attitude toward methods, and more tolerant because mutual tolerance among researchers is vital for a pluralism of methods. Psychological phenomena are complex and can best be understood by using different methods (Mayrhofer and Hutmacher, 2020). However, to get things working, tolerance must actively be lived. Of course, much depends on our own willingness as researchers but also on the system's arrangements. Psychology could be more colorful, and we could all have more fun if we were to be more committed to such a methodology. We hope our commentary has offered a view on methods that both new and established researchers in psychology will find attractive.

## Author Contributions

SZ: writing and lead. LL: writing. Both authors contributed to the article and approved the submitted version.

## Conflict of Interest

The authors declare tha the research was conducted in the absence of any commercial or financial relationships that could be construed as a potential conflict of interest.

## Footnotes

^1^It is interesting to note that, as Hacking pointed out in the Foreword of the fourth edition of *Against Method*, Feyerabend himself preferred the term “Dadaist” for various reasons. Thus, in a letter to Imre Lakatos, Feyerabend wrote that he hoped that “the reader will remember me as a flippant Dadaist and not as a serious anarchist.”
